# Costs and Epidemiological Changes of Chronic Diseases: Implications and Challenges for Health Systems

**DOI:** 10.1371/journal.pone.0118611

**Published:** 2015-03-17

**Authors:** Armando Arredondo, Raul Aviles

**Affiliations:** 1 Senior Researcher, National Institute of Public Health, Cuernavaca, Morelos, Mexico; 2 Associated Researcher, National Institute of Public Health, Cuernavaca, Morelos, Mexico; University of Calgary, CANADA

## Abstract

**Background:**

The need to integrate economic and epidemiological aspects in the clinical perspective leads to a proposal for the analysis of health disparities and to an evaluation of the health services and of the new challenges which are now being faced by health system reforms in middle income countries.

**Objective:**

To identify the epidemiological changes, the demand for health services and economic burden from chronic diseases (diabetes and hypertension) in a middle income county.

**Methods:**

We conducted longitudinal analyses of costs and epidemiological changes for diabetes and hypertension in the Mexican health system. The study population included both the insured and uninsured populations. The cost-evaluation method was used, based on the instrumentation and consensus techniques. To estimate the epidemiological changes and financial consequences for 2014–2016, six models were constructed according to the Box-Jenkins technique, using confidence intervals of 95%, and the Box-Pierce test.

**Results:**

Regarding epidemiological changes expected in both diseases for 2014 vs. 2016, an increase is expected, although results predict a greater increase for diabetes, 8–12% in all three studied institutions, (p < .05). Indeed, in the case of diabetes, the increase was 41469 cases for uninsured population (SSA) and 65737 for the insured population (IMSS and ISSSTE). On hypertension cases the increase was 38109 for uninsured vs 62895 for insured. Costs in US$ ranged from $699 to $748 for annual case management per patient in the case of diabetes, and from $485 to $622 in patients with hypertension. Comparing financial consequences of health services required by insured and uninsured populations, the greater increase (23%) will be for the insured population (p < .05). The financial requirements of both diseases will amount to 19.5% of the total budget for the uninsured and 12.5% for the insured population.

**Conclusions:**

If the risk factors and the different health care models remain as they currently are, the economic impact of expected epidemiological changes on the social security system will be particularly strong. Another relevant challenge is the appearance of internal competition in the use and allocation of financial resources with programs for other chronic and infectious diseases.

## Introduction

Chronic diseases present new challenges for health systems and require an integrated approach. The tendency for their incidence to increase has not been solved by the enhanced effort in providing treatments or by assigning economic resources that are necessary for these treatments. The idea prevails that the increase in the incidence of chronic diseases will continue [1].

In the context of health care reforms, the challenge of ensuring equity, efficiency, effectiveness and accessibility is directly related to the quality and quantity of the health services to be delivered, and also to the financial feasibility of generating these services [2]. Economic analysis, particularly of the cost of health service delivery as a function of changes in demand, is a relevant tool for monitoring health service performance [3].

Increases in health care costs, the need for increased investment, lack of financial support for health care users, and the urgency to change the resource allocation methods, have raised several questions among decision-makers, mainly among public policy planners and executives. Health care demand is increasing, and the high cost of alternatives during this transition period has become a heavy burden that national governments are trying to reduce [4–5].

With respect to health conditions, we need to take as a reference point trends in the epidemiological profile of developing countries proposed by epidemiological transition theory. This theory assumes that trends in morbidity and mortality correspond to certain changes in the frequency of both communicable and non-communicable diseases [6–7]. Even when cardiovascular diseases and accidents have made increasing demands on health care, infectious diseases are still among the ten main causes of morbidity and mortality in Latin American countries [8]. However, changes in the epidemiological profile in these countries will greatly impact the financing and production of services required for chronic illnesses [9].

From the economic standpoint, the changing epidemiological and demographic profiles in middle-income countries imply an increase in health care demand for costly conditions such as chronic or degenerative diseases and accidents, which will compete with resources allocated to treat infectious diseases [10–11]. Therefore, it is important to determine health care priorities and devise a strategy for optimal allocation, utilization and organization of financial resources within the health care system [12–13].

Allocation of resources to services for chronic diseases will affect those for infectious diseases; therefore, the financial requirements for services needed in the short- and medium-term must be known so that resources are allocated to each institution. In this sense, the health planning process regarding the production and financing of health care services will require the incorporation of clinical, epidemiological and economic indicators [14–15].

The present study aimed to determine epidemiological changes and case management costs of diabetes and hypertension, the two main demands on health services in Mexico, within the three most important public institutions: the Ministry of Health (SSA), the Mexican Social Security Institute (IMSS) and the Institute for Social Security and Services for State Workers (ISSSTE). According to data from the 2013–2018 National Health Program, the Mexican public health system provides care for 90% of the population and is mainly formed by the three health institutions that were included in this study[16]. It is important to highlight the fact that the IMSS provides services for the insured population and covers 45% of the population, the ISSSTE serves the insured population of state workers and covers approximately 5% of the population, and the SSA serves the uninsured population, which is 40% of the general population. The remaining 10% are served by the private sector [17].

Regarding the economic and epidemiological burden of chronic diseases, we present and discuss new results and challenges for health systems in middle income countries like Mexico. The main challenges are found in the epidemiological changes expected for the 2014–2016 period, as well as in the financial requirements to produce health services for the two studied diseases in the insured and uninsured populations. Finally, a discussion and conclusions are presented to identify implications, challenges and the financial consequences of the changes in demand, as well as to justify and guide investment in different health care services for diabetes and hypertension.

## Methods

Evaluative research was carried out, based on a longitudinal design to determine costs, epidemiological changes and financial requirements to deliver health care for diabetes type 2 and hypertension for the 2014–2016 period. Considering that the economic impact of cases that are being treated for the health system was measured; demand case concerns only the number of cases that request services and are under treatment and annual monitoring at each institution. The expected annual demand for health care services for diabetes and hypertension was calculated from a time series analysis using data from new cases and total cases for both diseases annually, depending on trends in incidence and prevalence by type of institution. The analysis was based on the total annual cases observed in the last 18 years in each study institution. The population base of the study included 1,268,912 patients with diabetes mellitus or hypertension, which were medically diagnosed in years prior to the study: 614 211 with diabetes and 654 701 with hypertension. This information was obtained from the statistics bulletin on health impairment of the National Health System [18]. The basic protocol of this project was reviewed and approved by the Committee on Health Research of the National Council of Science and Technology. The data on the number of observed cases were obtained from the annual report of epidemiological statistics for diabetes and hypertension [19], during the 1996–2013 period (Table 1). This is anonymous information that is used for the purpose of analysis.

**Table 1:** Annual total cases for diabetes and hypertension in Mexico reported per year during period 1996–2013.

YEARS	CASES OF DIABETES	CASES OF HYPERTENSION
1996	249 274	403 502
1997	312 892	440996
1998	336 967	490 850
1999	264 811	405687
2000	287 180	400 693
2001	294 198	411892
2002	315 498	428 730
2003	380 322	498 365
2004	404 562	558129
2005	397 387	519298
2006	394 360	521486
2007	404 770	521 159
2008	396 374	526484
2009	426 802	552530
2010	460 032	589 078
2011	487 125	591 575
2012	523 496	595 196
2013	579 391	639 203

Source: SSA. Reporte Anual de Casos de Enfermedades Crónicas. Sistema Único de Información para la Vigilancia Epidemiológica. Dirección General de Epidemiología. SSA. Mexico DF. 1996–2013: 1–2.

The three-year period was chosen for the study because, for chronic diseases, projections of more than 3 years may generate uncertainty and hence are not recommended [20]. The studied institutions belong to the public health sector of the Mexican health system: SSA (services for the uninsured population), and IMSS/ISSSTE (health care services for the insured population). The annual demand for health care services for hypertension and diabetes was calculated from the number of treated cases of diabetes and hypertension, adjusted by type of institution, as given in the 1996–2013 health impairment statistics bulletin of the National Health System [19].

Direct costs of health care services were obtained from the management of standardized cases, adjusted by type of institution. The standardization and adjustment for type of institution, was performed with the application of a discount rate of 2% annually, as economic recommendation for institutions generating services., based on the cost of annual average case handling and cost of inputs by type of institution [21]. The cost-evaluation method was designed according to an instrumentation technique that identified production and supply functions for each case management. Five formats were used to establish costs per production function, which were concentrated in cost-evaluation matrices, according to disease and institution. The format model was designed in a spreadsheet that included information to identify disease and type of institution. This spreadsheet is organized in 6 columns to determine the average cost of case management, including the following columns: input type, unit of measure, unit cost, quantity, equation depreciation and cost of managing annual average case (S1 Appendix). Case management was defined for an average case, with the corresponding adjustments made for each institution using the straight-line depreciation method for the case of infrastructure, furniture, equipment and instrumental.

For each disease and event to be evaluated, management of the average case was defined, based on the disease’s natural history and the results of shadow study reviews. The stages of the natural history of the disease considered were primary and secondary stage (health promotion early detection, diagnosis, timely treatment and limitation of damage and prevention of complications. Importantly, the stage of tertiary prevention regarding the management of major complications for both diseases was not consider. The ‘Shadow Study’ consisted of an observational study based on records of times and movements for the process of searching, obtaining and following the medical care provided to patients with hypertension and diabetes in each institution. The selection of patients was based on simple random sampling by selecting 50 patients under control and annual monitoring that came to the health unit in the months of January and June 2013, for a total of 100 patients per institution. This sample size was based on the criteria for shadow studies where it is recommended to follow up a number limited of cases observed in a particular period (between 5–10% of total patients attending medical control for a month) [22]. The point of view of a group comprised of expert clinicians and administrators was considered in order to obtain a homogeneous opinion on how to manage each case; these experts were selected in accordance with their medical specialty and experience (more than 10 years of experience in the management of patients with hypertension or diabetes in one of the target institutions). The definitions refer to the health care demand of hospital or ambulatory services, according to each disease.

In order to determine the financial requirements for the 2014–2016 period, a time series was done for the 1996 to 2013 period. The study population included all users who required services for the annual management of diabetes or hypertension in public institutions. A probabilistic model was estimated by the Box-Jenkins technique [23], using the Stat Graphics software with a confidence interval of 95%, p <.05, (S2 Appendix). In view of recommendations of similar studies [24–25], the following were included as variables in the models: trends in new cases of diabetes and hypertension in the last 15 years, trends in amounts invested in health promotion and disease prevention for uninsured vs. insured population, changes in the politics of access to health services, changes in the allocation of resources for health, and decentralization of the financing and production of services of the hypertension and diabetes programs.

To calculate the financial consequences of changes in demand by type of institution, in optimal scenarios, an inflationary index projected to 2014–2016 was developed and applied, based on the Banco de Mexico price index for consumers [26]. The accumulated inflation index was applied to the annual cost of management of each case per institution. In spite of the limitations associated with quantitative and economic projection, adjustment using the accumulated inflation index is the approach recommended by the Banco de Mexico for all prognoses of economic impact on health services. In this sense the financial requirements by type of institution was determined multiplying number of cases expected for case management costs at a rate of discount of 2% and applying an econometric inflation adjustment index.

## Results

The national annual average cost of case management per patient in US dollars was $707 for diabetes and $544 for hypertension. The cost results obtained in the present study can be used to establish minimum and maximum ranges of financing requirements for each disease at the three most important health care institutions in Mexico. The cost of a diabetes hospital case ranged from $699 to $748, with the lowest cost being at the SSA and the highest at the IMSS. The same tendencies were observed for the costs of ambulatory cases. In patients with hypertension, costs ranged from $485 to $622. In contrast to the result for diabetes, the lowest cost was for hypertension patients treated at the ISSSTE, while the highest cost was at IMSS.

**Fig 1:**
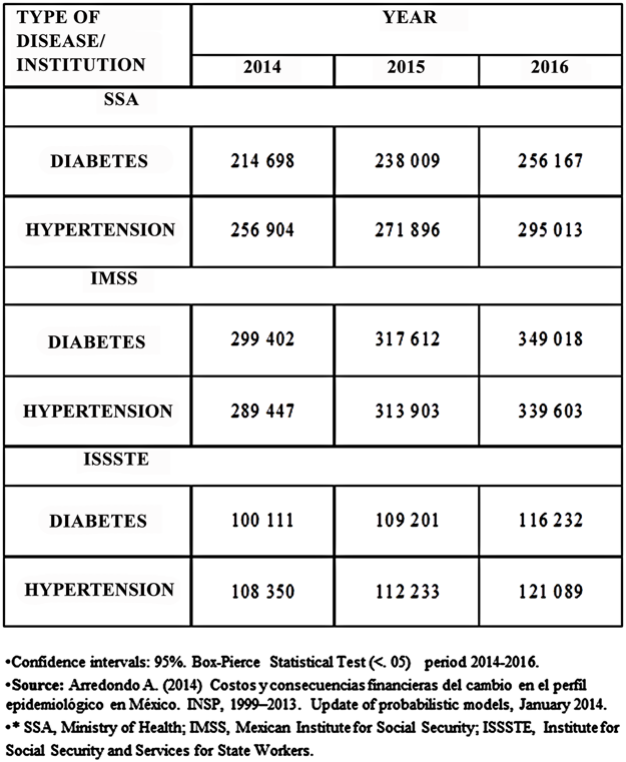
Expected Cases for the period 2014–2016 by type of disease and institution.

Regarding the effect of the expected epidemiological changes on the demand for hospital and ambulatory services for 2014–2016, the results for both diseases are shown in Fig. 1. In both cases, an increase is expected, although the results show a greater increase for diabetes. As shown in Fig. 1, the increase tends to be higher for the insured population than for the uninsured. In the case of diabetes, the increase was 41469 cases for uninsured population (SSA) and 65737 for the insured population (IMSS and ISSSTE). We should emphasize that the projection period was limited to 3 years because previous studies have advised against using periods greater than 3 years since they may cause uncertainty in budgeting.

**Fig 2:**
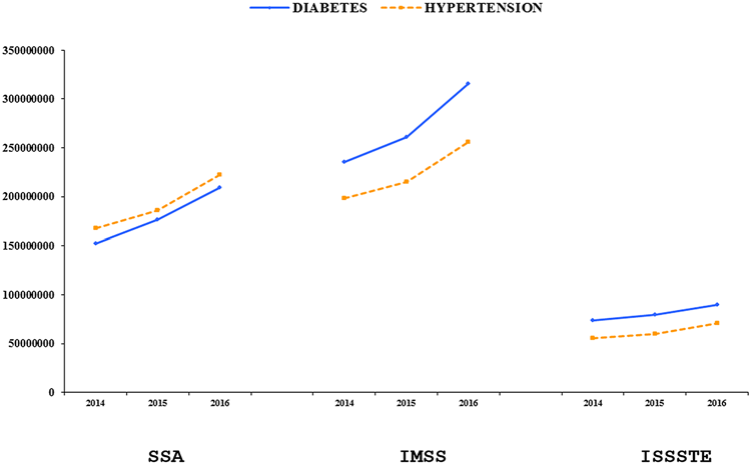
Costs from epidemiological changes expected to diabetes and hypertension in Mexico for the period 2014–2016 (in US $).

Fig. 2 shows trends in the economic resources in US$ needed to finance the minimum service demanded by the population. To cover service demand by diabetes patients, IMSS requirements will increase from to $235,150,330 to $315,889,211 (increase of 80,738,881, representing an increase of 40% over the period); for the ISSSTE they will increase from $73,476,468 to $89,849,963; and for the SSA, from $152,167,207 to $209,224,397. To cover service demand by hypertension patients, IMSS requirements will increase from $198,037,835 to $255,592,010; for the ISSSTE they will increase from $55,177,237 to $71,061,079; and for the SSA, from $167,784,002 to $222,032,684. Costs to satisfy health service demands by patients with hypertension in these three institutions showed the same trend as for patients with diabetes, although it is important to note that for the insured population, hypertension patients require less than half the resources considered necessary for diabetes patients. The range of growth rate for the period is between 35–45% for both diseases. Another important finding is that in the case of the uninsured population, epidemiological and economic trends are higher for hypertension than for diabetes (Fig. 2).

The institution for the insured population was found to have the highest average direct costs per case managed, as well as the highest economic impact of global management of diabetes and hypertension for 2014–2016. Financial requirements for health care services for both diseases represent 19.5% of the total budget assigned to the uninsured population, and 12.5% of that allocated to the insured population. If the risk factors and the different ambulatory and hospital care models remain more or less as they are in the three studied institutions, the financial burden will be higher for the IMSS, followed by the SSA and the ISSSTE. On the other hand, according to the results of our study, the insured population will account for 65% of the total financial burden for diabetes and hypertension health care, with the uninsured population accounting for the remaining 25%.

## Discussion

The validity of our results was confirmed, from an epidemiological standpoint, by reference to trends established during the previous 19 years. The models used in our projections were similar to those used in the statistics bulletin of the National Health System for 1996–2013. On the other hand, the number of expected cases follows similar trends to those projected by the International Diabetes Federation for some Latin American countries, highlighting as an example: 9,340,000 million cases for Brazil; 8,723,000 million for Mexico; 2,135,000 for Colombia; 1,608,000 for Argentina[27]. Case management costs obtained in the present work are within the range reported for the management of diabetes and hypertension in previous studies at the national and international levels [28–29].

The largest requirement for financial resources is expected in the insured population. This can be explained not only by an increase in demand due to epidemiological changes, but also by the increased inflationary index expected for 2014–2016. We must also stress that, in the case of the uninsured population, the predicted amount represents not only a lower financial requirement, but also reflects the fact that the increase in required financial resources is much more conservative than in the insured population.

The findings reflect important differences between the studied institutions, regarding the management of diabetes and hypertension. Differences in management costs among the institutions can be explained by differences in the costs of inputs, the way in which each institution combines the inputs to provide the health services required, and the quality of service provided.

As to the effects of the observed epidemiological changes on the demand for health care services for 2014–2016, a tendency towards an increase in the costs is expected, although the increase is greater for the insured population than for the uninsured. As health care reform progresses, the cost of delivering services to patients with hypertension and diabetes, who require only hospital care, will be higher than the cost of delivering services to patients with most infectious diseases who require hospital and ambulatory care [30]. The further the epidemiological transition progresses from infectious to chronic or degenerative disease, the greater will be the financial difficulty in satisfying health care demand for diseases such as diabetes and hypertension [31].

Respect to the differences in expected costs for the insured health subsystem (IMSS and ISSSTE) vs. the uninsured (SSA), the differences in results are mainly due to 3 reasons: The costs of supplies for social security institutions are higher, the population seeking care for hypertension is larger in uninsured care centers and there are differences in quality standards of care and of supplies in each institution.

With regard to the epidemiological changes expected for both diabetes and hypertension and their effects on service demand, it is important to mention that the number of expected cases during 2014–2016 refers to those under long-term control. This number was estimated from those under control from 1996 to the present in the top three health care institutions in Mexico [32]. The costs refer to the average annual management cost for ambulatory and hospital care, without considering severe complications.

Direct management cost differences between ambulatory and hospital care settings are clear indicators of the current development of health care systems. These correspond to significant differences in human resource costs, standards and quality of care, and the kind of production functions and raw materials needed to deliver such services. The observed differences in indirect costs are explained by variations between institutions and whether or not patients are insured [33].

The evidence on changes in costs and healthcare services required for chronic diseases can be used as a reference for the allocation of resources directed for health care services on hypertension or diabetes by different types of public institution [34]. For example, if the institutions know the financial requirements for each diseases, each institution could then target in a more effective and efficient way the resources allocation for this chronic diseases. The estimated financial requirements constitute the fundamental basis for strategic planning. Indeed, given the financial consequences of the expected epidemiological changes, not only is it essential to invest greater financial resources, but the implementation of health prevention strategies also becomes necessary [35].

From the perspective of the epidemiological and economic burden of chronic diseases, health systems in middle-income countries face great challenges. Their health systems were designed and organized to respond mainly to communicable diseases. Moreover, we must emphasize that in addition to the effects of epidemiological changes and trends in constant incremental costs of care, the problem is further complicated by some features of the health system and population. Indeed from the health system, there is resistance and institutional barriers they face when implementing primary care programs; from the side of the people, it is particularly difficult to target programs and interventions to reduce the effect of some social determinants of disease chronic, such as lifestyles, socio-economic status, education, etc.

To increase research on costs and financial consequences of changes in the epidemiological profile, a cost monitoring system should be established that will allow us to update this information so that it may be adjusted for inflation. We also recommend that the autoregressive probabilistic models be updated yearly, and that it would be beneficial to introduce a new variable specifying the geopolitical region. This is because evidence has shown that the northern and central regions of Mexico have epidemiological profiles that are very similar to those of developed countries, whereas the profiles of the southern region are more like those found in developing countries [36]. On the other hand, it is noteworthy that the results of epidemiological changes in our study with constant incremental trends are similar to those observed for diabetes and hypertension on the recent results of national surveys of health [37–38]

On the limitations of our results is necessary to highlight some aspects. First, on the epidemiological analysis of the frequency of cases of diabetes and hypertension, we only included cases that applied health services for diagnosis, treatment and control of disease in major health institutions in Mexico. In this sense, the analysis does not include cases ignoring be sick of diabetes or hypertension, or even knowing who are ill can not access health services for different reasons. Second, the analysis of time series under the Box-Jenkins method, its main limitation is required to have very good quality of information in the records of new cases and cumulative cases for a minimum period of 15 years. In our study delimit the record is 18 years (1996–2013), because before 1996 quality standards in the register were not of good quality. Third, the “Shadow Study” could have as its main limitation the failure to have the revision of 100% of patients who sought care services for diabetes or hypertension. However we must emphasize that compared to other methods such as Delphi technique or the technique of consensus, this method of qualitative analysis is what gives greater certainty regarding the verification of inputs used for production function.

## Conclusions

The results from this study can be used to determine where to invest in the health care system,. Estimations of financial requirements are basic information for strategic planning. In accord with other studies [39–41], the financial consequences of the expected epidemiological changes are not only a basis and justification for more investment in diabetes and hypertension management, but also for the allocation of more resources to the prevention of these conditions, thus minimizing and controlling disease and lessening the economic burden on health care services. With regard to the implications and challenges for the health system, we would like to conclude with the following statements:

1. Controlling costs and increasing demand for services for chronic diseases requires strategic programs for timely detection and prevention of these diseases and their complications. Certainly, this is one of the biggest challenges to face from a health system based on a biomedical model focused on the healing of communicable diseases with little actions in detection and prevention of chronic diseases.
2. The development of economic indicators would enable the design of patterns of resource allocation based on efficiency criteria with regard to clinical, epidemiological, economic and administrative aspects. Each institution could develop models for the distribution of resources in accordance with the changes in costs and epidemiological factors expected in future years.
3. The treatment of both chronic diseases should be approached from an integrated perspective including economic, and epidemiological aspects. In other words, an integrated approach to the problem of chronic diseases requires the development of indicators of expected epidemiological changes, economic requirements and demands for health care services to these diseases.
4. We should point out that financial pressure will not only depend on epidemiological changes by type of disease, but also on the type of population to be served. For example, in the case of insured patients at the IMSS, the demand for services for diabetes is much less than the demand for hypertension. Health care needs for patients with diabetes at the IMSS are almost the same as those at the SSA; however, they are much greater than the corresponding service needs for insured patients with diabetes at the ISSSTE.
5. In terms of the financial pressure generated from both the epidemiological changes and changes required to implement new health programs, the challenges for health planning will be different for the three institutions considered here. For example, the predicted financial consequences suggest the need to redesign and invest more in programs for health promotion and prevention, in order to reduce demand for hospital services and promote treatment of diabetes and hypertension in an ambulatory setting.
6. The greatest challenge will be for the social security services (IMSS and ISSSTE) because, since their creation, these services have received the greatest amount of funding for treatment and rehabilitation programs, but have invested very little in prevention programs. In practice, at all levels of decision-making in the social security institutions there is some barriers and difficulties to investing more money in health promotion and prevention of disease, despite the body of evidence showing the high costs involved in curing and rehabilitating patients with chronic health problems.
7. The repercussions of all of the above will have a greater relevance for health services for the uninsured population in terms of resource allocation. Indeed, in Latin American countries, traditionally the population that is outside of the formal economy and that does not have social security tends to neglect the problems of hypertension and diabetes due to a lack of monitoring programs for them. It has only been recently that these countries have begun to implement reforms in which health programs are designed exclusively for the prevention, management and control of patients with diabetes and/or hypertension. These services could form part of the “universal health insurance” that the present national health systems are implementing in Latin American countries. In this way, more technical parameters will be incorporated into the decision-making process for the efficient allocation and use of resources addressed for chronic disease like diabetes and hypertension.

## Supporting Information

S1 Appendix Model format to determine production costs by function (example for medical visit). (DOCX)

S2 Appendix Results on probabilistic models. (DOCX)
