# Supplementary material for: Costs and Epidemiological Changes of Chronic Diseases: Implications and Challenges for Health Systems
Source: PLoS One. 2015 Mar 17;10(3):e0118611. doi: 10.1371/journal.pone.0118611 (PMC4364072; doi:10.1371/journal.pone.0118611)
Supplement: S1 Appendix — (DOCX) [file pone.0118611.s001.docx]

***S1 Appendix: Model format to determine production costs by function ( example for medical visit)***

*INSTITUTION-------------------------- DISEASE-----------------------------------------------------------------*

| TYPE OF INPUT | MEASURE UNIT | UNIT COST | QUANTITY BY CASE MANAGEMENT | AVERAGE COST | EQUATION OF DEPRECIATION | TOTAL COST |
| --- | --- | --- | --- | --- | --- | --- |
| HUMAN RESOURCES |  |  |  |  |  |  |
| EQUIPMENT AND FURNITURE |  |  |  |  |  |  |
| DRUGS |  |  |  |  |  |  |
| DIAGNOSTIC STUDIES  ETC ...  ETC ...  INFRASTRUCTURE  MAINTENANCE SERVICES  GENERAL SERVICES  TOTAL COST |  |  |  |  |  |  |
