# Supplementary material for: Costs and Epidemiological Changes of Chronic Diseases: Implications and Challenges for Health Systems
Source: PLoS One. 2015 Mar 17;10(3):e0118611. doi: 10.1371/journal.pone.0118611 (PMC4364072; doi:10.1371/journal.pone.0118611)
Supplement: S2 Appendix — (DOCX) [file pone.0118611.s002.docx]

**S2 Appendix: Results on probabilistic models.**

Taking into account that the same method was used for both diseases, only the rationale for each step of the analysis employed for the case of diabetes is shown below.

Historical data were monthly numbers of diabetic cases collected over the period 1996-2013. These monthly numbers of cases, denoted as Y1, Y2,...,Y96, showed an increasing trend throughout the study period resulting in a slope coefficient of the trend analysis that was significantly different from zero (t = -.12). The data also showed a seasonal pattern with an increasingly higher peak each year, with the variable seasonal factor as well. Data were log-transformed to reduce the observed asymmetry. The natural logarithm of the number of cases is denoted by Y1,Y2,...,Y96.

It can be observed that the series is nonseasonal due to the slow decrease in the self-correlation function. After applying several seasonal and nonseasonal transformations, it was inferred that the seasonal time series can be explained by the transformation Zt = Yt - Yt-1 - Yt-12 + Yt-13, one seasonal and one nonseasonal differencing. Upon analyzing the self-correlation functions, it seems that the self-correlation function cuts the borderline of statistical significance of zero difference after lag 12. Some peaks are observed for smaller lags, indicating the need to include a seasonal average movement operator with some self-regressive operator and/or average movement operator of a smaller order. Based on the analysis of both functions, the following models were proposed:

MODEL 1. Seasonal average movement operator, order 1

 −  +  −  

MODEL 2. Seasonal average movement operator, order 1, and nonseasonal average movement operator, order 1

 −  +  −   −  +   

MODEL 3. The same as the model 2, but including the mean

MODEL 4. Seasonal self-regressive operator, order 1

 =  +  

MODEL 5. Nonseasonal self-regressive operator, order 1 and seasonal self-regressive operator, order 1

 =  +   +   −   

To select among several possible models, it was necessary to estimate the parameters of each and examine their properties. Model 1, which included only the seasonal average movement operator, fits the logarithms of the data with reasonably good results and a self-correlation different from zero in the lag 1, thus indicating that the model may be improved by adding a self-regressive and/or nonseasonal average movement operator.

Besides the seasonal average movement operator, model 2 included the nonseasonal average movement operator, which showed quite significant improvement in the standard deviation value that decreased from .4798 to .38007, and no nonzero self-correlation of residuals. The discussion on model 3 is carried out after discussing predictions. Models 4 and 5, which included seasonal and nonseasonal self-regressive operators, were also tested and were discarded because they did not meet adequacy conditions (substantially greater values of the Box-Pierce Chi-square and standard deviation than in the other two models). The model that adequately depicted the series to estimate the cases in diabetes and hypertension was the following:

 =  +  −   −  +   

with the prediction equation:

 =  +  +   −   +  + 

Therefore, the proposed model was model 3, which besides average movement and seasonal average movement operators, includes the mean estimator and variables mentioned in the methodology section. The outcomes of this model were better than those obtained using model 2 (lower standard error) whereas the t value for the mean estimator indicates that the trend is negative and significantly different from zero ( S2 Table).

**S2 Table: Statistical results of models 2 and 3.**

| **Variables** | **Model 2** | **Model 3** |
| --- | --- | --- |
| Number of nonseasonal differences | 1 | 1 |
| Number of seasonal differences | 0 | 0 |
| Number of Parameters | 2 | 3 |
| Estimator A | .2579 (2.43) | .7689 (4.01) |
| Estimator B | -.2723 (-2.54) | 0.5536 (2.23) |
| Estimator C | 0.8599 | 0.9123 |
| Box-Pierce Chi-square | 22.25 | 14.67 |
| Standard deviation | 24.22 | 26.9 |
| Nonzero correlations | 0 | 0 |
